# Long-term study reveals central European aerial insectivores as an unusual group of hosts that harbor mostly helminths that are unable to complete life-cycles in the nesting quarters of their hosts

**DOI:** 10.1186/s13071-022-05636-6

**Published:** 2023-01-26

**Authors:** Jiljí Sitko, Petr Heneberg

**Affiliations:** 1 Moravian Ornithological Station, Comenius Museum, Prerov, Czech Republic; 2 Third Faculty of Medicine, Charles University, Prague, Czech Republic

**Keywords:** Aerial insectivores, Diptera, Helminths, Migration, Population dynamics, Trematoda, Urban birds

## Abstract

**Background:**

Central European aerial insectivores are long-distance migrants that winter in sub-Saharan Africa. Most of them employ the fly-and-forage migrating strategy and differ in their food composition. The composition and structure of helminth component communities of these hosts are poorly understood, and information regarding seasonality and long-term changes is unavailable.

**Methods:**

From 1963 to 2022, we analyzed the population trends of helminths in five aerial insectivore species. Namely, we examined *Apus apus*, *Hirundo rustica*, *Delichon urbicum*, *Riparia riparia*, and *Ficedula albicollis*; all originated from the Czech Republic.

**Results:**

We identified central European aerial insectivores as hosts that are parasitized mostly by helminths that cannot complete their life-cycles in the nesting quarters of their hosts. This phenomenon is unknown in other bird host species. In contrast, only a single dominant trematode species that completes its life-cycle locally colonized the central European aerial insectivores. All other dominant species of Trematoda, all Nematoda, and all Acanthocephala were dependent on intermediate hosts unavailable in the nesting quarters of the examined bird hosts. Surprisingly, these helminths transmitted from winter quarters or migratory routes were diverse, and many of them were abundant in terms of both prevalence and intensity of infection. The helminth component communities of aerial insectivores were dynamic systems. During the study period, three species became new and regularly encountered members of helminth fauna of examined hosts, and other species gradually increased or decreased their intensity of infection. In contrast to other groups of bird hosts, the dominant helminth species of aerial insectivores did not experience local extinctions or rapid population losses.

**Conclusions:**

The analysis of helminths of five central European aerial insectivores revealed component communities that heavily rely on completing host–parasite cycles at migration routes or wintering grounds. The composition of the analyzed component communities changed dynamically during the 60-year-long study period, but there was no evidence of large-scale declines in abundance or prevalence.

**Graphical Abstract:**

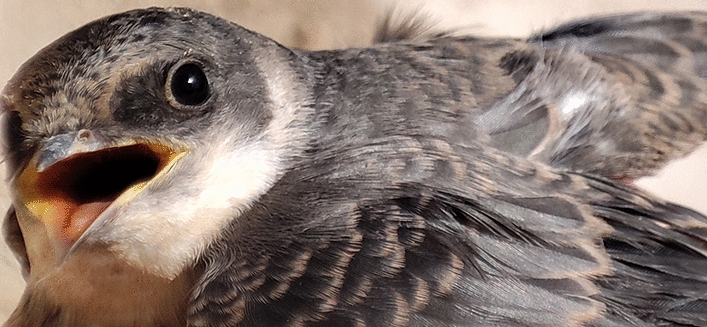

**Supplementary Information:**

The online version contains supplementary material available at 10.1186/s13071-022-05636-6.

## Background

Central European swifts, swallows, and martins are long-distance migrants that winter in sub-Saharan Africa [1]. Despite all being aerial insect feeders, they differ in hunting strategies and in the location of their winter quarters. The commonly nesting species in central Europe consist of the common swift [*Apus apus* (Linnaeus, 1758)], barn swallow (*Hirundo rustica* Linnaeus, 1758), house martin [*Delichon urbicum* (Linnaeus, 1758)], and bank swallow [*Riparia riparia* (Linnaeus, 1758)]. Despite all four species forming abundant populations, little is known regarding their helminths, including their population trends and host preferences. All of these species are sensitive to bad weather conditions. Their sensitivity leads to the delivery of large amounts of aerial insectivores to wildlife rescue centers for rehabilitation, deaths of juveniles in their nests, and adults being hit by cars near larger water bodies [2].

Central European swifts and swallows differ in their food composition. From the parasitological point of view, the essential difference is the presence of Orthoptera in the food of swallows and martins [3, 4], while this order is absent from the food of common swifts [5–7]. The ingestion of Orthoptera is directly related to the presence of *Diplotriena* nematodes in the food of Orthoptera-consuming species. The common swift typically preys on aerial plankton, i.e., a wide variety of insects and arachnids [5–7]. The barn swallow typically preys on large dipterans (Tabanidae, Syrphidae, and Muscidae) [3, 8]. The decline in these groups of insects, resulting from changes in farming practices, is thought to cause declines in reproductive success and quality of the offspring produced by barn swallows [9, 10]. The studies that focused on the presence of livestock concluded that barn swallows feed more on large Diptera and Coleoptera when livestock are present and on smaller prey, mostly Hymenoptera, when livestock are absent [11]. Coleoptera now prevail even in the food of barn swallow nestlings [12]. The diet of the house martin consists mainly of Hemiptera: Aphididae (particularly in the spring months), Diptera: Bibionidae (particularly in the summer months), Diptera: Cyclorrhapha, and, to some extent, Hymenoptera: Parasitica & Formicoidea [13]. The diet of the bank swallow consists mainly of Diptera (68% of fecal samples—mainly Chironomidae, much less Anthomyiidae, Dolichopodidae, and Sphaeroceridae), Coleoptera (36%), Hymenoptera (14%), Hemiptera (12%), and Lepidoptera (9%) [14]. The diet of the fifth species included in this study, the collared flycatcher [*Ficedula albicollis* (Temminck, 1815)], consists of Diptera, Coleoptera (23% each), Arachnida (14%), Lepidoptera (13%), and Hymenoptera (12%)—these data apply to nestlings [15]. Similar data were reported in other studies examining collared flycatcher nestlings [16], but data from adults are unavailable.

The helminths of central European insectivorous birds have rarely been studied. Most data are available regarding the common swift. The morphology of *Lyperosomum clathratum* (Deslongschamps, 1824) was extensively addressed by Sitko [17], while the representatives of *Brachydistomum* from various swift species were subject to revision by the same author earlier [18]. *Plagiorchis maculosus* (Rudolphi, 1802) and *P. elegans* (Rudolphi, 1802) (*cirratus*) were examined in common swifts and barn swallows by Odening [19]. Ottó Sey examined 12 common swifts and 15 barn swallows for their trematodes in Hungary. The common swifts were positive only for *Brachydistomum salebrosum* (Braun, 1901) and *L. clathratum*. The barn swallows were positive for *Prosthogonimus ovatus* (Rudolphi, 1803) and *P. maculosus* [20]. In Moldova, 107 house martins and 22 bank swallows were studied in 1958–1962 by Shumilo [21], who found 18 and 7 species of helminths, respectively.

In the present study, we address the composition and structure of helminth component communities of four obligate central European aerial insect feeders (common swift, barn swallow, house martin, and bank swallow) and compare it with the helminth component community of a facultative aerial feeder (collared flycatcher). When the weather permits, the latter species behaves as an aerial insect feeder, but after rain it also feeds on the ground; therefore, it encounters Acanthocephala, *Leucochloridium*, and *Urogonimus* from molluscs and *Porrocaecum* from earthworms. As the data collection period reached 60 years, we further tested for any long-term changes in component communities of helminths in the analyzed insectivorous birds. Adults might harbor helminths acquired when away from breeding grounds, and the food composition of adults and nestlings may differ. Therefore, we analyzed the component communities of first-year birds and those of adult females or males separately.

## Methods

From 1963 to 2022, we examined 261 individuals of *A. apus*, consisting of 104 adult males, 77 adult females, and 80 birds in the first calendar year (1Y). We further examined 447 individuals of *H. rustica*. The dataset included 269 adult males, 134 adult females, and 40 1Y birds. We examined 151 individuals of *D. urbicum*, namely, 66 adult males, 35 adult females, and 50 1Y birds. We examined 186 individuals of *R. riparia*, namely, 108 adult males, 73 adult females, and five 1Y birds. We examined 87 individuals of *F. albicollis*, namely, 56 adult males, 27 adult females, and four 1Y birds. The numbers of hosts examined in individual decades of the study period are shown in Table 1.

**Table 1:** Numbers of host individuals that were examined in the present study and their distribution within individual decades

Species	Study period, number of individuals					
	1963–1980	1981–1990	1991–2000	2001–2010	2011–2020	2021–2022
*Apus apus*	7	17	62	69	98	8
*Hirundo rustica*	73	19	49	234	66	6
*Delichon urbicum*	53	17	9	29	43	0
*Riparia riparia*	4	55	47	75	5	0
*Ficedula albicollis*	0	4	19	46	16	2

We sorted the examined birds according to their age into 1Y birds (born in the calendar year when they were examined) and adults (birds in their second calendar year of life or older) [22, 23]. We also recorded the sex of the adults. All of the examined birds originated from the eastern and southern Czech Republic (48.7° N–49.80° N, 13.3° E–18° 30′ E). We obtained the dead birds before they were prepared for the Comenius Museum collection (Přerov, Czech Republic). The birds consisted primarily of wounded or injured individuals, most of which were sacrificed in rescue stations due to untreatable wounds. The swallows and martins originated mainly from the Nové Mlýny reservoirs, where enormous numbers of swallows and martins are hit by cars passing through the route on the top of the dam between the central and lower reservoirs. Concerning birds provided by the rescue stations, they included only individuals untreated with antihelmintic agents before their sacrifice. Governmental and local authorities authorized our long-term research; our most recent permit was issued by the Ministry of the Environment of the Czech Republic on August 3, 2009 (No. 11171/ENV/09-747/620/09-ZS 25).

We performed full-body necropsies and examined the subcutaneous tissue, body cavity, esophagus, stomach, intestines, cloaca, bursa of Fabricius, liver, gall bladder, spleen, lungs, trachea, bronchus, air sacs, kidneys, and oviducts using a stereomicroscope. We fixed the helminths in 70% ethanol, stained them with borax carmine, transferred them through an alcohol series to xylene, and mounted them in Canada balsam as described previously [24]. We recorded the abundance and species richness of helminths in each examined host individual. We stored representative specimens in the Comenius Museum collections (Přerov, Czech Republic). We published the new host–parasite records from the examined datasets in our previous studies and had already used some of the analyzed helminths for molecular analyses [25–28]. The nomenclature follows the Fauna Europaea database [29] and recently published reclassifications. For details concerning the life-cycles of the examined helminths, refer to Sitko et al. [30].

We calculated the basic characteristics of the analyzed component communities (mean frequency of infection and helminth load). We also calculated helminth species-specific mean relative prevalence (the proportion of host individuals infected by helminths) and mean intensity of infection (the number of trematode individuals per host calculated over all individuals that were positive for the respective trematode). We computed rarefaction curves to interpolate the trematode species richness data [31]. To estimate the true trematode species richness, we calculated the Chao1 estimator corrected for unseen helminth species [32, 33]. We compared the helminth species richness using the presence/absence-based Sørensen similarity index and the abundance-based Bray–Curtis similarity index. We tested for differences in helminth diversity among the analyzed component communities using the Shannon diversity *t*-test (comparing values of the Shannon *H* index with a bias correction term proposed by Poole [34] of two abundance datasets while assuming equal sampling conditions).

We correlated the frequency of infections, the overall intensity of infections, and species-specific data on the abundance of individual helminth species with the year when the respective host bird individuals were received. To avoid a bias that could be caused by differences in prevalence and infection intensities in juveniles and adults and to avoid possible sex-related bias, we evaluated the adult females, adult males, and 1Y birds for each species separately (we did not evaluate long-term changes in 1Y *R. riparia* or 1Y *F. albicollis* due to the low number of examined birds in these categories). To comment on changes in time, we split the dataset into four time periods. The first was 1963–1990, which corresponds to specific conditions associated with the socialist regimen that was present in the study region. We split the follow-up period into 1991–2000, 2001–2010, and 2011–2022.

We correlated the frequency of infections, the helminth load, and species-specific data on the abundance of individual helminth species with the month when the respective host bird individuals were received. To avoid a bias that could be caused by differences in prevalence and infection intensities in juveniles and adults and to avoid possible sex-related bias, we evaluated the adult females, adult males, and 1Y birds for each species separately (we did not evaluate long-term changes in 1Y *R. riparia* or *F. albicollis* due to the low number of examined birds in these categories).

We employed one-way permutational multivariate analysis of variance (PERMANOVA) to identify the differences among adult males, females, and 1Y bird hosts. We further used non-metric multidimensional scaling (NMDS) to analyze the effects of explanatory variables (age, sex, month, and year). We performed all calculations in SigmaPlot 12.0, EstimateS 9.1.0, and PAST 2.14. Data are shown as the mean ± standard error (SE) unless stated otherwise.

## Results

### Helminth component communities of aerial insect feeders

We collected a total of 4730 individuals belonging to 33 species of helminths. The helminth load differed among the host species by nearly one order of magnitude (Table 3). We analyzed host species-specific component communities in *A. apus* (2076 individuals, 16 helminth species), *H. rustica* (1150 individuals, 15 helminth species), *D. urbicum* (1055 individuals, 11 helminth species), *R. riparia* (346 individuals, 3 trematode species), and *F. albicollis* (103 individuals, 9 helminth species) (Additional file 1: Table S1). The analyzed assemblages consisted of four species of Acanthocephala, six species of Nematoda, 21 species of Trematoda, and Cestoda, which we did not identify to the species level (Table 2). Regarding the species identity of Cestoda, *H. rustica* hosts two species of Cestoda (and perhaps an additional rare species, which we have never found in the study region). We were able to identify Cestoda to the species level in well-preserved material, but the identification was inconclusive in degraded material.

**Table 2:** Host-specific component communities of helminths found in the course of the present study

Helminth species	Host species, number of individuals found				
	*Apus apus* (*n* = 261)	*Hirundo rustica* (*n* = 447)	*Delichon urbicum* (*n* = 151)	*Riparia riparia* (*n* = 186)	*Ficedula albicollis* (*n* = 87)
Acanthocephala:					
*Apororhynchus paulonucleatus* Hohlova & Cimbaluk, 1971	0	3	0	0	0
*Mediorhynchus micracanthus* (Rudolphi, 1819)	1	0	0	0	2
*Mediorhynchus papillosus* Van Cleave, 1916	0	11	1	0	0
*Plagiorhynchus (Prosthorhynchus) cylindraceus* (Goeze, 1782)	0	0	0	0	8
Cestoda gen. sp.	680	325	462	276	22
Nematoda:					
*Acuaria attenuata* Rudolphi, 1819	0	9	11	0	5
*Capillaria* (*Tridentocapillaria*) *hirundinis* (Rudolphi, 1819)	1	2	0	0	0
*Diplotriaena henryi* Blanc, 1919	1	0	0	0	0
*Diplotriaena obtusa* (Rudolphi, 1802)	0	107	4	14	0
*Physaloptera apodis* Vuylsteke, 1954	1	0	3	0	0
*Porrocaecum ensicaudatum* (Zeder, 1800)	0	0	0	0	2
Trematoda:					
*Brachydistomum olssoni* (Railliet, 1900)	881	0	0	0	0
*Brachydistomum salebrosum* (Braun, 1901)	191	0	0	0	0
*Brachylecithum donicum* (Issaitschikov, 1909)	19	20	0	0	0
*Collyriclum faba* (Bremser in Schmalz, 1831)	0	136	0	0	0
*Cortrema magnicaudata* (Bykhovskaya-Pavlovskaya, 1950)	0	102	2	0	0
*Laterotrema vexans* (Braun, 1901)	5	0	0	0	0
*Leucochloridium perturbatum* Pojmanska, 1969	0	0	0	0	35
*Lyperosomum api* Sitko & Heneberg, 2023	1	0	0	0	0
*Lyperosomum baskakowi* (Iwanitsky, 1927)	0	0	0	0	3
*Lyperosomum clathratum* (Deslongschamps, 1824)	232	0	0	0	0
*Lyperosomum tenori* Sitko & Heneberg, 2023	0	14	15	0	0
*Mosesia amplavaginata* Oshmarin, 1970	0	11	4	0	0
*Mosesia monedulae* Oshmarin, 1970	3	0	0	0	0
*Plagiorchis elegans* (Rudolphi, 1802)	2	2	0	0	0
*Plagiorchis maculosus* (Rudolphi, 1802)	51	287	543	2	22
*Posthovitellum contribulans* (Braun, 1901)	6	0	0	0	0
*Posthovitellum ripariae* (Schumilo, 1965)	0	10	8	0	0
*Prosthogonimus ovatus* (Rudolphi, 1803)	1	0	0	0	0
*Skrjabinus kalmikensis* Skrjabin & Issaitschikow, 1927	0	0	2	0	0
*Stromitrema koshewnikowi* (Skrjabin & Massino, 1925)	0	111	0	0	0
*Urogonimus macrostomus* (Rudolphi, 1802)	0	0	0	0	4

**Fig. 1:**
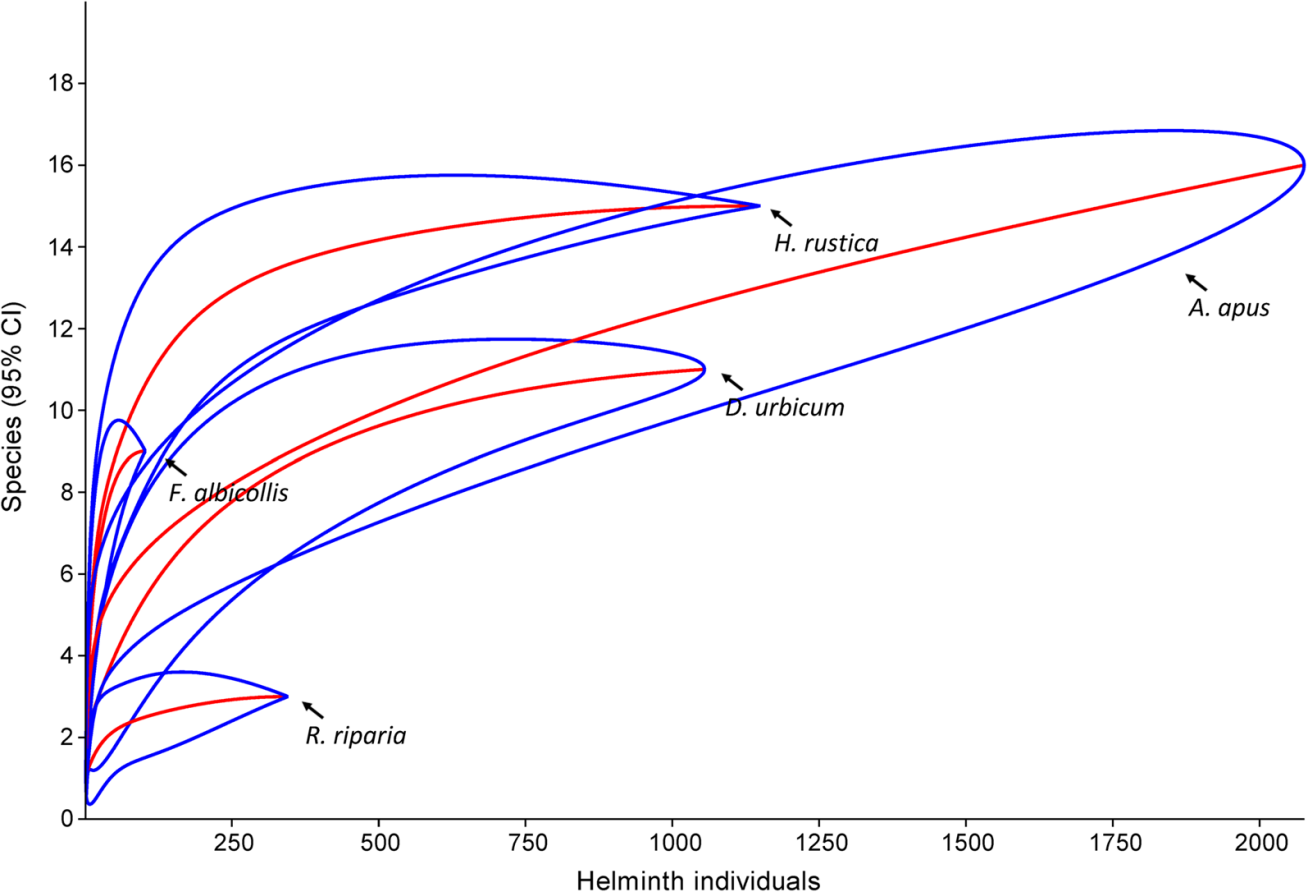
Rarefaction curves of the component communities of *A. apus*, *H. rustica*, *D. urbicum*, *R*. *riparia*, and *F. albicollis* sampled in the course of the present study

Rarefaction analysis indicated that the helminth component communities of four of the five bird host species were nearly complete; only the component community of *A. apus* was sampled incompletely, although we examined 261 individuals of this species (Fig. 1). The Chao1 estimates of helminth species richness in the analyzed component communities were 23.5 (95% CI 17.3–58.5) species in *A. apus*, 15.0 (95% CI 15.0–16.4) species in *H. rustica*, 11.0 (95% CI 11.4–12.1) species in *D. urbicum*, 3.0 (95% CI 3.0–3.8) species in *R. riparia*, and 8.0 (95% CI 8.0–9.4) species in *F. albicollis* (Table 3).

**Table 3:** Diversity indices and outcomes of the Shannon diversity *t*-test

Variable	Host species				
	*Apus apus* (*n* = 261)	*Hirundo rustica* (*n* = 447)	*Delichon urbicum* (*n* = 151)	*Riparia riparia* (*n* = 186)	*Ficedula albicollis* (*n* = 87)
Number of species	16	15	11	3	9
Number of individuals	2076	1150	1055	346	103
Helminth load (mean ± SE)	8.0 ± 0.8	2.6 ± 0.4	7.0 ± 0.7	1.9 ± 0.3	1.2 ± 0.5
Dominance	0.309	0.183	0.457	0.089	0.781
Evenness	0.254	0.467	0.232	0.409	0.642
Equitability	0.504	0.719	0.391	0.186	0.798
Fisher alpha	2.36	2.44	1.71	0.45	2.37
Berger–Parker	0.424	0.283	0.515	0.954	0.340
Shannon diversity *t*-test (*t*; *df*; *P*)					
*Apus apus*		12.1; 5511.8; < 0.001	−13.3; 2120.5; < 0.001	−15.9; 2113.6; < 0.001	3.8; 115.4; < 0.001
*Hirundo rustica*			24.7; 2845.8; < 0.001	18.2; 5034.0; < 0.001	1.4; 388.2; 0.17
*Delichon urbicum*				7.1; 2462.7; < 0.001	−8.9; 128.0; < 0.001
*Riparia riparia*					−10.4; 376.2; < 0.001

The species composition and diversity differed among the analyzed species. The Shannon diversity *t*-test revealed significant differences in all pairwise comparisons except for *H. rustica* and *F. albicollis* (Table 3). The species of the component communities of *H. rustica* and *D. urbicum* overlapped the most in terms of the number of species (Sørensen similarity index 0.720; Table 4), while the similarity among all other species combinations was much lower. Abundance data also suggested the highest similarity between component communities of *H. rustica* and *D. urbicum* (Bray–Curtis similarity index 0.594). In addition, the Bray–Curtis index differentiated between the component communities of swallows and martins (all having Bray–Curtis index > 0.4), swallows/martins and swifts (all having Bray–Curtis index at 0.24–0.33), and swallows/martins/swifts and the collared flycatcher (Bray–Curtis index at 0.04–0.12; Table 4). In absolute numbers, the component communities of *H. rustica* and *D. urbicum* shared nine helminth species, the component communities of *H. rustica* and *A. apus* shared five helminth species, and all other component communities shared only two or three species (Table 4).

**Table 4:** Comparison of the diversities of the analyzed component communities

Variable	Host species				
	*Apus apus* (*n* = 261)	*Hirundo rustica* (*n* = 447)	*Delichon urbicum* (*n* = 151)	*Riparia riparia* (*n* = 186)	*Ficedula albicollis* (*n* = 87)
Sørensen similarity index					
*Apus apus*		0.333	0.222	0.211	0.250
*Hirundo rustica*			0.720	0.353	0.273
*Delichon urbicum*				0.429	0.316
*Riparia riparia*					0.364
Bray–Curtis similarity index					
*Apus apus*		0.247	0.328	0.235	0.041
*Hirundo rustica*			0.594	0.406	0.079
*Delichon urbicum*				0.419	0.085
*Riparia riparia*					0.123
No. of helminth species found in both host species					
*Apus apus*		5	3	2	3
*Hirundo rustica*			9	3	3
*Delichon urbicum*				3	3
*Riparia riparia*					2
No. of helminth species unique for the first/second compared host species					
*Apus apus*		10	8	1	5
*Hirundo rustica*	11		2	0	5
*Delichon urbicum*	13	6		0	5
*Riparia riparia*	14	12	8		6
*Ficedula albicollis*	13	12	8	1	

Alpha diversity, quantified using Fisher alpha, was very low in *R. riparia* (0.45) and several times higher in the other four host species (1.71–2.44). The dominance values differed strongly among the analyzed hosts and ranged between 0.09 (*R. riparia*) and 0.78 (*F. albicollis*). The evenness differed among the analyzed species, with high values obtained in *F. albicollis* (0.64), whereas component communities of *A. apus* and *D. urbicum* were associated with low evenness (0.25 and 0.23, respectively). Equitability ranged between 0.19 (*R. riparia*) and 0.72 (*H. rustica*). The Berger–Parker index ranged between 0.28 (*H. rustica*) and 0.95 (*R. riparia*) (Table 3).

### Long-term changes in helminth infections

The long-term changes were significant in only several abundantly present helminth species. All four *Acanthocephala* spp. were absent from birds that were examined in 1963–1990. In the three later periods, they were rarely, but regularly, spotted at mean intensities of infection not exceeding two helminth individuals per host individual and prevalence not exceeding 2%. The Cestoda gen. sp. were abundantly present in all four time periods. Their prevalence gradually decreased from 50.6% in 1963–1990 to 32.0% in 2011–2022. Correspondingly, the intensities of infection decreased from 5.6 to 3.9 helminth individuals per host individual.

Of the six *Nematoda* spp., only *Diplotriaena obtusa* (Rudolphi, 1802) was present in all four time periods, and its prevalence increased from an initial 2.0% to a peak of 6.0% in 2011–2020. Correspondingly, the infection intensity by this species increased from 1.8 in 1963–1990 to 3.2 helminth individuals per host individual in 2011–2022. The other Nematoda spp. were absent in 1963–1990 and 1991–2000, and four were only rarely spotted in the latter periods (prevalence < 0.5%, intensity of infection ≤ 3 helminth individuals per host individual). The exception was *Acuaria attenuata* Rudolphi, 1819, which we found for the first time in six *H. rustica* individuals and one *F. albicollis* individual in 2013, in four *D. urbicum* individuals and one *F. albicollis* individual in 2014, and two *H. rustica* individuals in 2015. We did not find any *A. attenuata* outside the 2013–2015 period. In 2011–2022, it reached a prevalence of 5.7% and an infection intensity of 1.8 helminth individuals per host individual.

Concerning the 21 *Trematoda* spp., only five species were present throughout the four examined periods. In contrast, 15 species were absent in 1963–1990, and nine were absent in 2011–2022. The constantly present species included *Brachydistomum olssoni* (Railliet, 1900), *B. salebrosum*, *L. clathratum*, *P. maculosus*, and *Stromitrema koshewnikowi* (Skrjabin & Massino, 1925). These species differed in their population trends. *Brachydistomum olssoni* is declining; the intensity of infection by this species decreased from 24.8 helminth individuals per host individual to only 7.5 helminth individuals per host individual in 2001–2010 and 8.8 helminth individuals per host individual in 2011–2022. In contrast, the intensity of infection by *B. salebrosum* gradually increased from an initial 7.0 helminth individuals per host individual to 25.5 helminth individuals per host individual in 2011–2022. The intensity of infection by *L. clathratum* (3.7 vs. 3.5 helminth individuals per host individual) and *P. maculosus* (4.6 vs. 5.5 helminth individuals per host individual) remained stable. The intensity of infection by *S. koshewnikowi* gradually decreased from 6.8 to 3.0 helminth individuals per host individual.

The Trematoda species that were absent in 1963–1990 were represented by rare infection events, and their prevalence did not exceed 1% in any of the follow-up periods, except for *Lyperosomum tenori* Sitko & Heneberg, 2023, *Posthovitellum contribulans* (Braun, 1901), *Mosesia amplavaginata* Oshmarin, 1970, and *Brachylecithum donicum* (Issaitschikov, 1909).

*Lyperosomum tenori* is a recently described species that we found to infect *H. rustica* and *D. urbicum*. We recorded it for the first time in *D. urbicum* in 1991; the follow-up records in *D. urbicum* were in 2010, 2012, 2014, and 2019 (one host individual each). In *H. rustica*, we found three *L. tenori*-positive host individuals in 2001; the follow-up records in *H. rustica* were in 2002, 2008, 2009 (two host individuals each), 2010, and 2014 (one host individual each). The mean intensity of infection reached 3.3 helminth individuals per host individual in 2011–2022.

*Posthovitellum contribulans* infected *A. apus*. We recorded it for the first time in *A. apus* in 1995; the follow-up records in *A. apus* were in 1998 and 1999 (one host individual each). The mean intensity of infection reached 2.0 helminth individuals per host individual. We did not find any *P. contribulans* outside the 1995–1999 period.

*Mosesia amplavaginata* infected *H. rustica* and *D. urbicum*. We recorded it for the first time in 1991 in one *H. rustica* and one *D. urbicum*. The follow-up records were in 2001 when we found another *H. rustica* and *D. urbicum* (one individual each) infected with this species. The mean intensity of infection reached 3.8 helminth individuals per host individual. We did not find any *M. amplavaginata* outside the 1991–2001 period.

*Brachylecithum donicum* infected *H. rustica* and *A. apus*. We recorded it for the first time in three individuals of *H. rustica* in 2001. In *A. apus*, we found the first *B. donicum*-positive host individual in 2005; the follow-up records in *A. apus* were in 2006, 2011, and 2018 (one host individual each). The mean intensity of infection reached 5.6 helminth individuals per host individual.

The Trematoda species that were absent in 2011–2022 were represented by rare infection events, and their prevalence did not exceed 1% in any of the follow-up time periods, except for *P. contribulans* and *M. amplavaginata*, which were also absent in 1963–1990 and are discussed above.

**Fig. 2:**
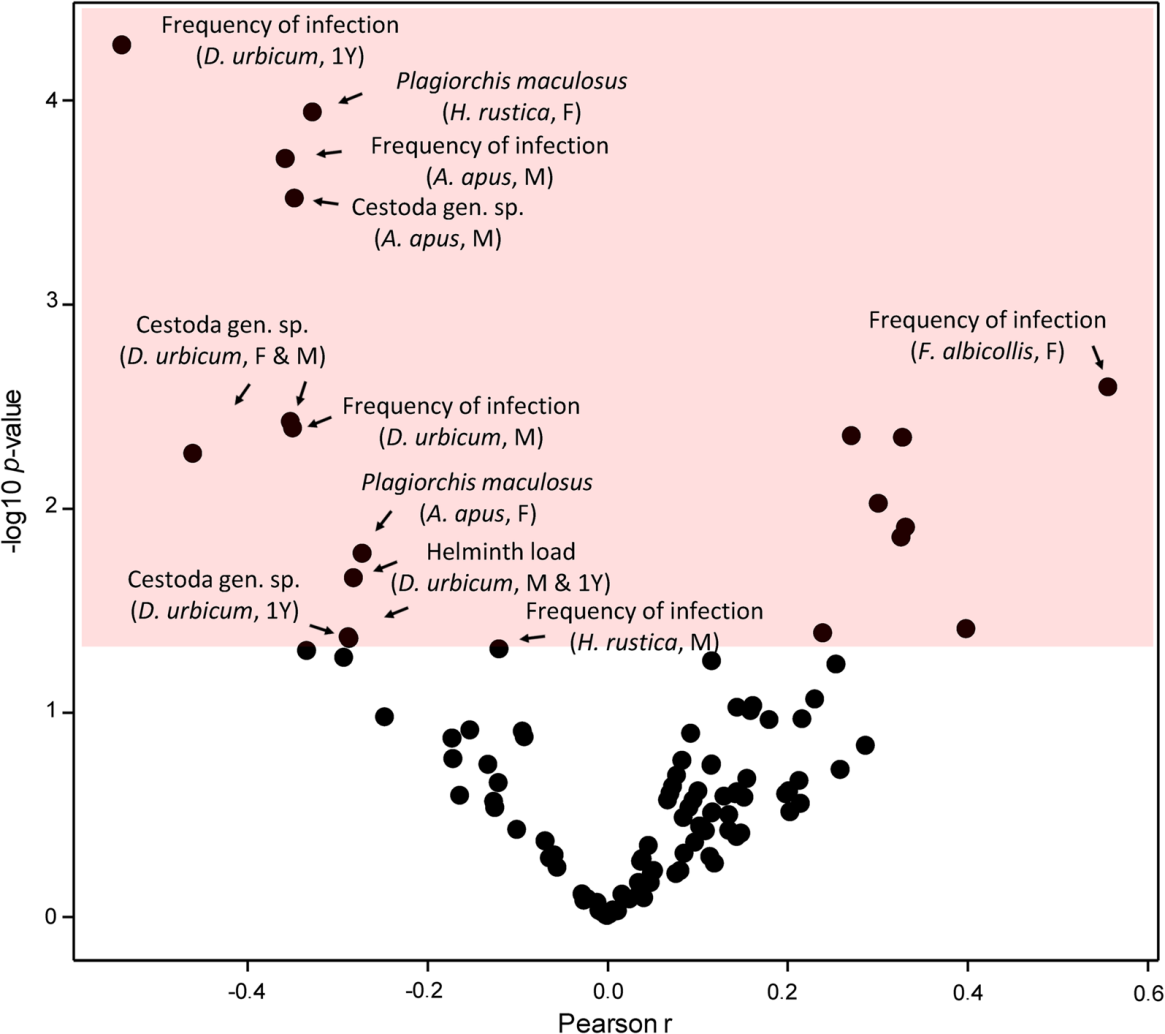
Volcano plot for Pearson correlations between the year when the bird host was acquired and the frequency of infections, the helminth load, and species-specific data on the abundance of individual helminth species. The color space indicates significant correlations (*P* < 0.05). Selected significant variables are labeled on plots

Often, the changes at the level of individual helminth species were insignificant. Nevertheless, the overall frequency of infection or helminth load changed (Fig. 2), indicating that the study was underpowered to detect long-term population trends in rare helminth species.

**Fig. 3:**
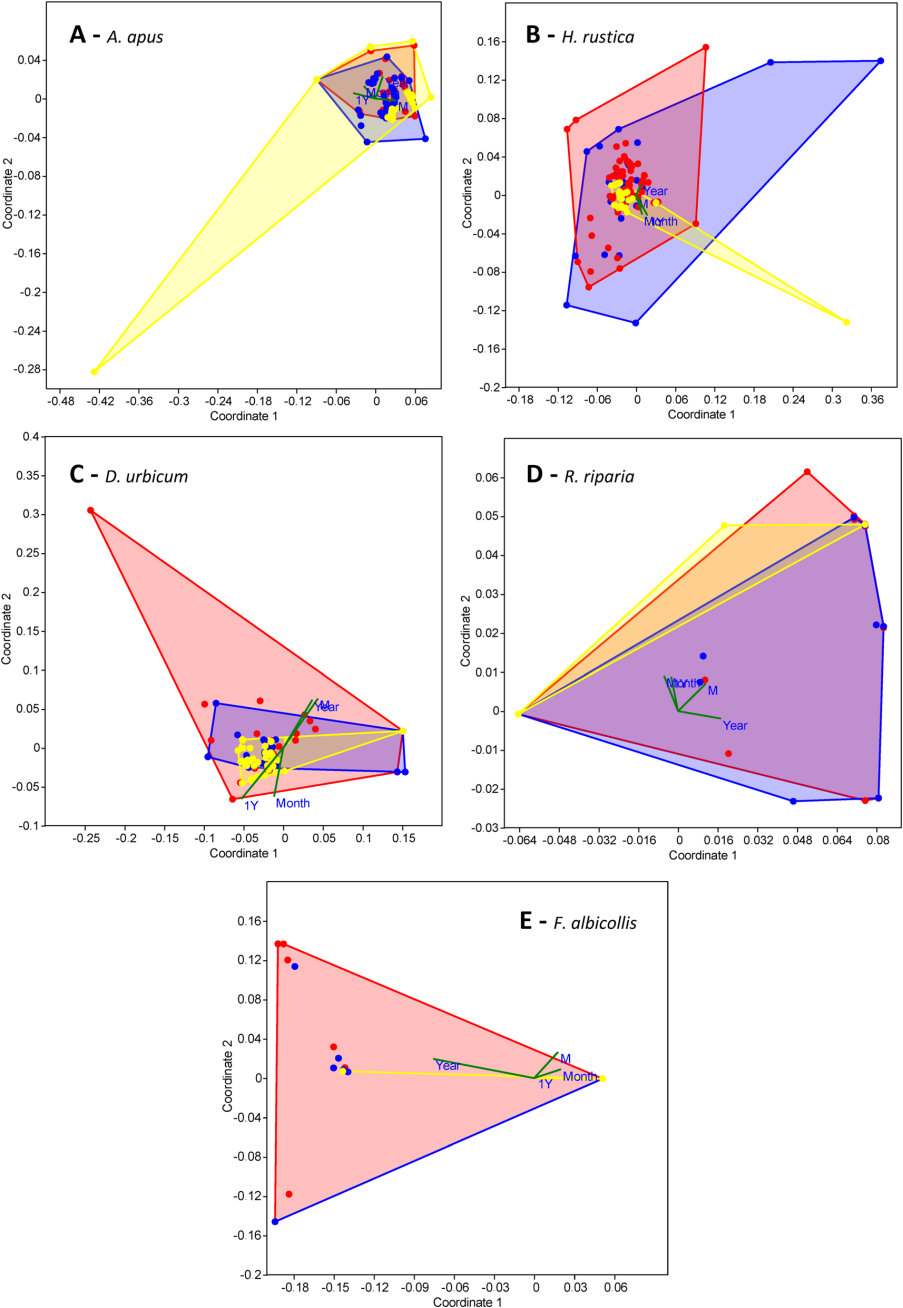
NMDS (Bray–Curtis distance measure) plots of the effects of environmental variables [age (adult M = 1, adult F = 0), sex (1Y = 1, adult = 0), month, and year] on the analyzed helminth component communities. **A** *Apus apus*, **B** *Hirundo rustica*, **C** *Delichon urbicum*, **D** *Riparia riparia*, **E** *Ficedula albicollis*. Yellow = 1Y birds, red = adult males, blue = adult females. Points show host individuals; convex hulls indicate host individuals of the same type

In *A. apus* females, the frequency of infection was not subject to changes over time, but in *A. apus* males the decline in the frequency of infection was significant (Pearson *r* = −0.36, *P* < 0.001). The frequency of infection decreased from 1.84 ± 0.16 species per host individual in 1963–2000 (*n* = 37) to 1.13 ± 0.11 species per host individual in 2011–2022 (*n* = 40). Interestingly, there was no such decline in 1Y birds (Pearson *r* = 0.18, *P* = 0.11). In *A. apus* females, the abundance of *P. maculosus* decreased over time (Pearson *r* = −0.27, *P* = 0.016); this trend was, however, absent in *A. apus* males (Pearson *r* = 0.10, *P* = 0.09), and this helminth species was absent in 1Y birds. In *A. apus* males, the abundance of Cestoda gen. sp. (Pearson *r* = −0.35, *P* < 0.001) decreased; this decline was not recapitulated in females or 1Y birds. The differences among component communities of adult males, adult females, and 1Y *A. apus* were significant [one-way PERMANOVA (Bray–Curtis distance measure): permutation *N* = 9999, total sum of squares 0.0098, within-group of squares 0.0093, *F* = 6.948, *P* < 0.001]. Subsequent pairwise comparisons revealed that the component community of 1Y birds differed significantly from both adult males and females (*P* = 0.0001, and *P* = 0.0009, respectively), and the differences between adult males and females were also significant (*P* = 0.015). Subsequent NMDS analysis confirmed that the composition of component communities overlapped (Fig. 3A).

In *H. rustica* females, the frequency of infection was not subject to changes over time, but in *H. rustica* males, the decline in the frequency of infection was significant (Pearson *r* = −0.12, *P* = 0.049). A similar trend was present in 1Y birds (Pearson *r* = −0.25, *P* = 0.11). In *H. rustica* females, the abundance of *P. maculosus* decreased over time (Pearson *r* = −0.33, *P* < 0.001), but this trend was absent in *H*. *rustica* males and 1Y birds. The differences among component communities of adult males, adult females, and 1Y *H. rustica* were significant [one-way PERMANOVA (Bray–Curtis distance measure): permutation *N* = 9999, total sum of squares 166.7, within-group of squares 164.5, *F* = 2.926, *P* = 0.006]. Subsequent pairwise comparisons revealed that the component community of adult females differed significantly from adult males (*P* = 0.014) and 1Y birds (*P* = 0.006), but the differences between adult males and 1Y birds were not significant (*P* > 0.05). Subsequent NMDS analysis confirmed that the composition of component communities overlapped (Fig. 3B).

In *D. urbicum* females and males, the frequency of infection decreased over time (Pearson *r* = −0.33, *P* = 0.05, and Pearson *r* = −0.35, *P* = 0.004, respectively). In females, the frequency of infection decreased from 1.50 ± 0.20 species per host individual in 1963–2000 (*n* = 14) to 1.08 ± 0.13 species per host individual in 2011–2022 (*n* = 13). In males, the frequency of infection decreased from 1.35 ± 0.14 species per host individual in 1963–2000 (*n* = 26) to 0.93 ± 0.11 species per host individual in 2011–2022 (*n* = 28). The decline in 1Y birds was even sharper (Pearson *r* = −0.54, *P* < 0.001). In 1Y birds, the frequency of infection decreased from 1.46 ± 0.09 species per host individual in 1963–2000 (*n* = 39) to 1.09 ± 0.16 species per host individual in 2001–2022 (*n* = 11). The helminth load remained stable in *D. urbicum* females but declined in males (Pearson *r* = −0.28, *P* = 0.02) and 1Y birds (Pearson *r* = −0.29, *P* = 0.04). More specifically, the declines affected Cestoda gen. sp. in *D. urbicum* females (Pearson *r* = −0.46, *P* = 0.005), males (Pearson *r* = −0.35, *P* = 0.004), and 1Y birds (Pearson *r* = −0.29, *P* = 0.04). The differences among component communities of adult males, adult females, and 1Y *D. urbicum* were significant [one-way PERMANOVA (Bray–Curtis distance measure): permutation *N* = 9999, total sum of squares 0.0082, within-group of squares 0.0071, *F* = 11.580, *P* < 0.001]. Subsequent pairwise comparisons revealed that the component community of 1Y birds differed significantly from adult males (*P* = 0.0001) and females (*P* = 0.0003), but the differences between adult males and females were not significant (*P* > 0.05). Subsequent NMDS analysis confirmed that the composition of component communities overlapped (Fig. 3C).

In *R. riparia* females and males, the frequency of infection increased over time (Pearson *r* = 0.24, *P* = 0.04, and *r* = 0.27, *P* = 0.004, respectively). In females, the frequency of infection increased from 0.52 ± 0.10 species per host individual in 1963–2000 (*n* = 40) to 0.52 ± 0.61 species per host individual in 2001–2022 (*n* = 33). In males, the frequency of infection increased from 0.40 ± 0.52 species per host individual in 1963–2000 (*n* = 63) to 0.78 ± 0.47 species per host individual in 2001–2022 (*n* = 45). The helminth load increased in *R. riparia* females (Pearson *r* = 0.30, *P* = 0.009), and trends in a similar direction were present in males (Pearson *r* = 0.16, *P* = 0.10). More specifically, Cestoda gen. sp. increased in abundance in *R. riparia* females (Pearson *r* = 0.32, *P* = 0.005), and similar trends were observed in males (Pearson *r* = 0.16, *P* = 0.09). The differences among component communities of adult males, adult females, and 1Y *R. riparia* were significant [one-way PERMANOVA (Bray–Curtis distance measure): permutation *N* = 9999, total sum of squares 0.0018, within-group of squares 0.0016, *F* = 9.819, *P* < 0.001]. Subsequent pairwise comparisons revealed that the component community of 1Y birds differed significantly from adult males (*P* = 0.002) and females (*P* = 0.004); the component communities of adult males and females were significantly different too (*P* = 0.0005). Subsequent NMDS analysis confirmed that the composition of component communities overlapped (Fig. 3D).

In *F. albicollis* females and males, the frequency of infection increased over time (Pearson *r* = 0.55, *P* = 0.003, and *r* = 0.33, *P* = 0.01, respectively). In females, the frequency of infection increased from 0.13 ± 0.11 species per host individual in 1963–2000 (*n* = 8) to 0.37 ± 0.11 species per host individual in 2001–2022 (*n* = 19). In males, the frequency of infection increased from 0.08 ± 0.07 species per host individual in 1963–2000 (*n* = 13) to 0.28 ± 0.08 species per host individual in 2001–2022 (*n* = 43). Similarly, the helminth load increased in both *F. albicollis* sexes (Pearson *r* = 0.40, *P* = 0.04, and *r* = 0.33, *P* = 0.01, respectively). There were no significant species-specific trends in *F. albicollis* helminths, which is directly related to their relatively low abundance in this host species. The differences among component communities of adult males, adult females, and 1Y *F. albicollis* were significant [one-way PERMANOVA (Bray–Curtis distance measure): permutation *N* = 9999, total sum of squares 0.00068, within-group of squares 0.00061, *F* = 4.974, *P* = 0.009]. Subsequent pairwise comparisons revealed that the component community of 1Y birds differed significantly from adult males (*P* = 0.024) and females (*P* = 0.015); the component communities of adult males and females were significantly different too (*P* = 0.033). Subsequent NMDS analysis confirmed that the composition of component communities overlapped (Fig. 3E).

### Seasonality of infections

**Fig. 4:**
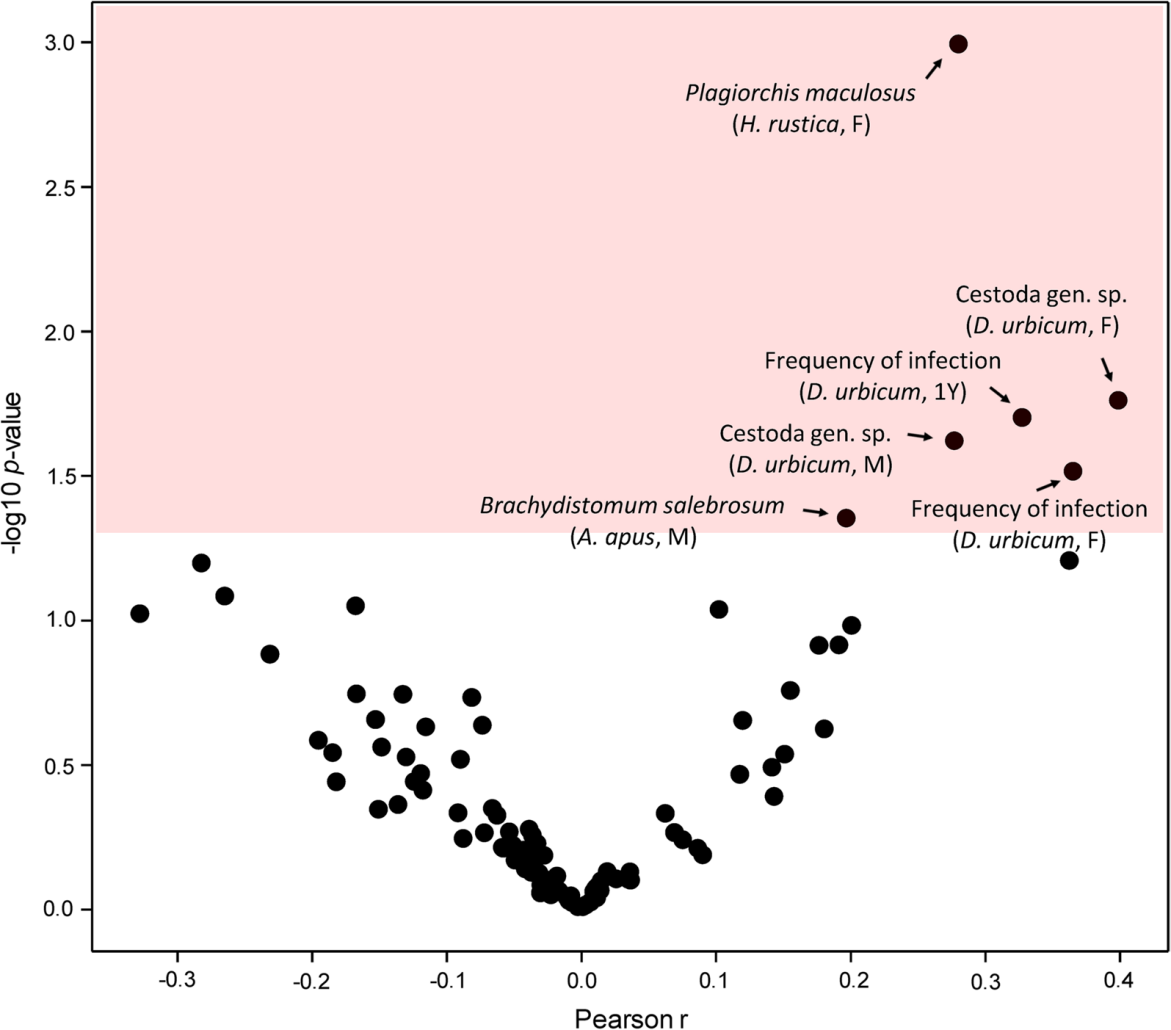
Volcano plot for Pearson correlations between the month when the bird host was acquired and the frequency of infections, the helminth load, and species-specific data on the abundance of individual helminth species. The color space indicates significant correlations (*P* < 0.05). Selected significant variables are labeled on plots

The frequency of infections and the helminth load did not display any significant correlations with the month when the respective host bird individuals were received except for the frequency of infections in adult females and 1Y *D. urbicum*. At the helminth species level, the seasonality was significant in only several abundantly present helminth species (Fig. 4).

In *A. apus* females, we did not identify any seasonality-related changes in helminth abundance or frequency of infection. In *A. apus* males, the abundance of *B. salebrosum* in the summer months was higher than that in the spring months (Pearson *r* = 0.20, *P* = 0.045). Nevertheless, this trend was not present in *A. apus* females, and this helminth was absent from 1Y birds.

In *H. rustica* females, the abundance of *P. maculosus* in the summer months was higher than that in the spring months (Pearson *r* = 0.28, *P* = 0.001); a similar trend was present in *H*. *rustica* males (Pearson *r* = 0.10, *P* = 0.09) but not in 1Y birds.

In females of *D. urbicum*, we found an increase in the overall frequency of infection (Pearson *r* = 0.37, *P* = 0.03); a similar trend was present in *D. urbicum* males (Pearson *r* = 0.20, *P* = 0.11), and even in 1Y birds (Pearson *r* = 0.32, *P* = 0.02). In females of *D. urbicum*, we also found an increase in the abundance of Cestoda gen. sp. (Pearson *r* = 0.40, *P* = 0.002); the abundance of Cestoda gen. sp. was also higher in the summer months than in the spring months in *D. urbicum* males (Pearson *r* = 0.28, *P* = 0.02) but not in 1Y birds.

In *R. riparia*, we found only a limited number of helminth species. Their changes were not significant in any of the sexes or ages. Similarly, there were no significant seasonality-related changes in helminths of *F. albicollis*.

### Helminth species absent in juvenile hosts

Many helminths of common swifts and swallows were absent in 1Y host individuals. The 1Y host individuals were associated with a low number of species, almost all of which were shared with the adults (except three species in *A. apus* and one in *F. albicollis*, but all these represented species with low prevalence in the examined hosts). An analysis of the similarities between component communities of adult and 1Y birds is shown in Table 5. The Acanthocephala and Nematoda were completely absent in juveniles. Four species of Trematoda were present in juveniles and therefore had locally available intermediate hosts. Another four species were absent in juveniles but are known from other central European bird hosts that are sedentary or short-distance migrants. The largest group of trematodes was represented by 13 species that were present only in adult host individuals, and that have no intermediate hosts known from central Europe (Table 5).

**Table 5:** Comparison of component communities in adult and 1Y host individuals

Variable	Host species				
	*Apus apus* (*n*_adults_ = 181; *N*_1Y_ = 80)	*Hirundo rustica* (*n*_adults_ = 403; *N*_1Y_ = 44)	*Delichon urbicum* (*n*_adults_ = 101; *N*_1Y_ = 50)	*Riparia riparia* (*n*_adults_ = 181; *N*_1Y_ = 5)	*Ficedula albicollis* (*n*_adults_ = 83; *N*_1Y_ = 4)
Species observed (adults)	13	15	11	3	7
Species observed (1Y)	5	3	2	1	1
Shared species (observed)	2	3	2	1	0
Chao shared species (estimated)	0	3.25	0	0	0
Sørensen similarity index	0.222	0.353	0.308	0.500	0.000
Bray–Curtis similarity index	0.230	0.241	0.794	0.312	0.000
Shannon diversity *t*-test (*t*; *df*; *P*)	13.8; 375.4; < 0.001	23.0; 200.0; < 0.001	8.3; 554.3; < 0.001	5.2; 292.0; < 0.001	14.7; 81.0; < 0.001

*Apus apus* had the highest intensity of infection by species that do not complete their life-cycle locally at the nest places of the definitive hosts among all the central European birds (Sitko, unpubl.). The swallows and martins were also infected by unusually high numbers of species that do not complete their life-cycles locally. Nevertheless, the intensities of infection by these species were much lower than those experienced in *A. apus*. In other insectivorous birds, including *F. albicollis*, which we included in the present study, the intensities of infection by species that do not complete their life-cycle locally were even lower.

However, it is unclear whether the helminths that complete their life-cycle locally differ in their abundance from species that do not complete their life-cycle locally. These species were abundant—when disregarding Cestoda, which we did not identify as species, the species that do not complete their life-cycle locally represented the second through seventh most abundant species in *A. apus*. Similarly, they represented the second through 11th most abundant species in *H. rustica* and the second through 10th most abundant species in *D. urbicum*. Note that in the three above host species, the only species more abundant than those that did not complete their life-cycle locally were outnumbered only by *P. maculosus*. We examined only several 1Y *R. riparia* and *F. albicollis*; therefore, the comparison is not available for these two host species. As specified below, the helminths that complete their life-cycle locally did not differ in trends of their prevalence and intensity of infection from species that do not complete their life-cycle locally. Some species that do not complete their life-cycle locally increased their prevalence and/or intensity of infection (*B. salebrosum*). In contrast, others decreased their prevalence and/or intensity of infection (*B. donicum* and *S. koshewnikowi*) or were present only during a narrowly defined period (*A. attenuata*).

## Discussion

We identified aerial insectivores as an unusual group of host species that harbor mostly helminths that cannot complete their life-cycles in the nesting quarters of their hosts. This phenomenon is unknown in other bird host species. Usually, helminths that do not complete their life-cycle locally are detected in migratory species but are rare relative to species with locally available intermediate hosts (e.g., [24, 35, 36]). In contrast, the central European aerial insectivores were colonized by only a single dominant trematode species that completed its life-cycle locally, and all other dominant species of Trematoda, all Nematoda, and all Acanthocephala were dependent on intermediate hosts that were not available in nesting quarters of the examined bird hosts. It was surprising that these helminths transmitted from winter quarters or migratory routes were both diverse, and many of them were abundant in terms of both prevalence and intensity of infection.

The prevalence and intensity of infection by helminths transmitted from winter quarters or migratory routes usually decrease in the course of the nesting season, and they are, therefore, more likely to be identified in the spring, shortly after the arrival of the respective host (e.g., [24, 35, 36]). However, we did not find such trends in helminths infecting the analyzed host species. The difference between other host species and aerial insectivores stems from the short time spent at their nest places. *Apus apus* arrive at their nest sites in early May and leave the nesting grounds in late July or the first days of August [1]. Therefore, they spend only 3 months at the nesting grounds compared with 9 months along the migration route and at winter quarters. In the migration and winter quarters, they appear to synchronize their movements according to changes in food availability, for which the index of changing greenness, such as the normalized difference vegetation index, can be used as a good proxy. During the migration and winter quarters, insect abundance peaks approximately 5 to 6 weeks after the start of rainfall [37]. Thus, the southward movement of the Intertropical Convergence Zone across the African continent from October to January is associated with high abundance and large concentrations of aerial insect prey [38]. In sub-Saharan Africa, swarming termites may constitute a dominant form of the prey of aerial insectivores [39]. The migration strategy of *A. apus* is of the fly-and-forage type [40]; therefore, insects from both the migration routes and winter quarters could serve as intermediate hosts of their helminths. The swallows and martins spend somewhat longer time at their nesting quarters. From the three analyzed swallow and martin species, the shortest time at nest places is spent by *R. riparia*, and most of them arrive at their nesting colonies during May and leave them gradually from late June to early September, often spending another month or two in large marshes in the Pannonian lowland before starting the migration itself. They use the fly-and-forage migration strategy during migration, with many stopovers [41]. The fifth species, *F. albicollis*, arrives in mid-April [42] and leaves the nesting grounds in August or September. In contrast to swifts, swallows, and martins, it is a nocturnal migrant, extending migration into the daytime when crossing the Sahara [43]. The species composition of prey captured by aerial insect feeders in their winter quarters is poorly understood [38, 39], which makes it challenging to determine the potential hosts of the helminths transmitted from the winter quarters and migration routes. The intermediate hosts are, therefore, unknown for many of these helminth species.

The absence of many helminths of common swifts and swallows in 1Y host individuals could be caused by differences in diet or by the absence of intermediate hosts at breeding quarters. The Acanthocephala were completely absent in juveniles. The intermediate hosts of *Mediorhynchus micracanthus* (Rudolphi, 1819) and *Mediorhynchus papillosus* Van Cleave, 1916 are Coleoptera. *Mediorhynchus* spp. develop in *Adesmia* (Tenebrionidae) [44]. *Adesmia* spp. are characteristic of desert habitats along the migration routes of examined definitive hosts. In the Czech Republic, we find *Mediorhynchus* spp. to be particularly abundant in adult (but not juvenile) great reed warblers *Acrocephalus arundinaceus* (Sitko, unpubl.). The intermediate hosts of *Plagiorhynchus* (*Prosthorhynchus*) *cylindraceus* (Goeze, 1782) are Isopoda (*Armadillidium*, and *Porcellio*). We speculate that *F. albicollis* does not provide its nestlings with isopods, and the adults become infected during spring or summer months when the weather is rainy. Some other local birds, such as Turdidae, have infected juveniles (Sitko, unpubl.). The intermediate hosts of *Apororhynchus paulonucleatus* Hohlova & Cimbaluk, 1971 are unknown.

Concerning Nematoda, we also did not find any in the juveniles. Nevertheless, most nematodes have locally available hosts, and their absence in juvenile birds likely stems from differences in the food composition of the juveniles and adults. *A. attenuata* develops in Orthoptera [45]. *Capillaria* (*Tridentocapillaria*) *hirundinis* (Rudolphi, 1819) has unknown intermediate hosts but is also present in sedentary central European bird species. *Diplotriaena henryi* Blanc, 1919 and *D. obtusa* develop in insects, likely locally [46]. *Physaloptera apodis* Vuylsteke, 1954 has a cosmopolitan distribution, but the intermediate hosts are unknown. *Porrocaecum ensicaudatum* (Zeder, 1800) also has a cosmopolitan distribution, developing in Lumbricidae. Therefore, we detected *P. ensicaudatum* only in *F. albicollis*.

Concerning Trematoda, *B. olssoni*, *Mosesia monedulae* Oshmarin, 1970, *P. elegans* and *P. maculosus* (intermediate hosts of *Plagiorchis* spp.: Insecta [47]), and *P. ovatus* (intermediate hosts: Odonata) were present in juveniles and therefore had locally available intermediate hosts. Of the species absent in juveniles, *Collyriclum faba* (Bremser in Schmalz, 1831) [48], *Leucochloridium perturbatum* Pojmanska, 1969 [28], *Prosthogonimus cuneatus* [26], and *Urogonimus macrostomus* (Rudolphi, 1802) [28] have intermediate hosts available locally and are commonly found in central European bird hosts that are sedentary or short-distance migrants. The remaining 13 species were only in adult host individuals, and have no intermediate hosts known from central Europe. In most of these trematodes, the intermediate hosts are unknown; only for *Posthovitellum* and *Laterotrema* are the intermediate hosts known to be Odonata [49–52]. The 13 southern species were *B. salebrosum* (191 individuals found), *B. donicum* (39 individuals), *Cortrema magnicaudata* (Bykhovskaya-Pavlovskaya, 1950) (104 individuals), *Laterotrema vexans* (Braun, 1901) (5 individuals), *Lyperosomum api* Sitko & Heneberg, 2023 (one individual), *Lyperosomum baskakowi* (Iwanitsky, 1927) (three individuals), *L. clathratum* (232 individuals), *L. tenori* (29 individuals), *M. amplavaginata* (15 individuals), *P. contribulans* (six individuals), *Posthovitellum ripariae* (Schumilo, 1965) (18 individuals), *Skrjabinus kalmikensis* Skrjabin & Issaitschikow, 1927 (two individuals), and *S. koshewnikowi* (111 individuals). Interestingly, the aerial insect feeders were not affected by the simplification of helminth communities that we reported previously in wetland birds [53] and the loss of previously dominant helminths in birds that use farmlands as their feeding habitats [54]. We observed declines in the frequency of infection and/or helminth load in *A. apus*, *H. rustica*, and *D. urbicum*. In contrast, the helminth load increased in *R. riparia* and *F. albicollis*. The first three host species are associated with urban environments. In contrast, the latter two host species typically occupy habitats outside urban areas (although *R. riparia* is already in the process of transition to municipal city centers, a process that is more advanced in other countries such as Spain [55]). There were changes associated with individual helminth species. Some, such as *A. attenuata*, *L. tenori*, and *B. donicum*, became new members of the helminth fauna of the examined host species. Others, such as *B. salebrosum*, gradually increased the intensity of infection. Another species, *S. koshewnikowi*, gradually decreased its intensity of infection. No helminth species were abundantly present in the 1960s–1980s and then locally extinct in any follow-up period. The decline in flying insect biomass is a problem affecting areas of high human density and activity, including the study region [56–61]. Surprisingly, it had only limited effects on the structure of helminth component communities of aerial insect feeders. However, this may correspond to the fact that the flying insect population trends in sparsely populated regions and wildlands are largely unknown [62], although these regions represent the migratory routes and winter quarters of the host species studied.

Further research is needed to confirm that the absence of helminth species in 1Y birds was not caused by differences in diet between the adult and juvenile birds and truly represented the absence of their intermediate hosts in the study area. As we pointed out in the Discussion, four helminth species lacking in 1Y host birds had the intermediate hosts available locally, whereas this evidence (either direct or indirect) was lacking for another 13 helminth species. The life-cycles of most of these 13 species are incompletely understood.

## Limitations

The present study relied on an opportunistic sampling design, where the carcasses of the six examined species consisted solely of wounded or injured individuals and therefore may not necessarily represent the helminths that would be present in birds of good health. We also cannot exclude the possibility that species with low prevalence escaped detection due to the limited number of examined host individuals. This applies particularly to the number of available 1Y individuals of *R. riparia* and *F. albicollis*, which prevented any detailed analyses of the helminth component communities of 1Y individuals of these two host species. The sample sizes also differed dramatically among the study periods; for example, *F. albicollis* individuals were absent in 1963–1980, and only four were examined in 1981–1990. These issues are difficult to resolve because the number of available host individuals is highly variable and depends on weather conditions, the willingness of the public to deliver injured individuals to rescue stations, and the cooperation of the rescue stations themselves. However, none of these influences affects the ability of the present study to explore the composition of dominant species within the analyzed component communities and to illustrate overall longitudinal trends in the analyzed component communities.

## Conclusions

We uncovered central European aerial insectivores as hosts that are parasitized mostly by helminths that cannot complete their life-cycles in the nesting quarters of their hosts. This phenomenon is unknown in other bird host species. In particular, *A. apus* is surprisingly frequently parasitized by species that develop in their winter quarters; the share of such species is higher than that of any other bird species. This result is likely related to the short time spent by *A. apus* at their nest sites. This anomaly somewhat corresponds only to other aerial insect feeders, the *Hirundinidae*. Although the number of helminth species that develop in winter quarters is somewhat higher in *Hirundinidae* than in *A. apus*, their prevalence was half of that found in *A. apus*. Beyond aerial insectivores, the other insectivorous birds host a much lower number of species that develop in winter quarters and their prevalence [30]. The helminth component communities of aerial insectivores were dynamic systems. During the study period, three species became new and regularly encountered members of their helminth fauna, and other species gradually increased or decreased their intensity of infection. In contrast to other groups of bird hosts, the dominant helminth species of aerial insectivores did not experience local extinctions or rapid population losses.

## Supplementary Information

**Additional file 1: Table S1.** Raw data. Listed are all examined *H. rustica*, *D. urbicum*, *R*. *riparia*, *F. albicollis*, and *A. apus* host individuals together with the abundance of all helminth species found. The age and sex of the analyzed hosts as well as the month and year when we acquired the respective host individual are indicated.

## Data Availability

Representative specimens of the helminths analyzed in this study are available in the collections of the Comenius Museum in Přerov. All data are available in the main text or the Additional file.

## Abbreviation

1Y: Bird in the first calendar year
